# Medical Journalism

**Published:** 1898-08

**Authors:** 


### "MEDICAL JOURNALISM."

UNDER the above title the *Boston Medical and Surgical Journal* prints in its editorial columns, July 22, 1898, an article that we need make no apology for reproducing in full. It contains much of interest to every medical editor and publisher, and every contributor to the medical press ought to read it carefully and ponder well its dignified sentiments and adopt its high moral tone.

Within the past few years the increase in number of medical weekly and monthly publications has been so enormous that all contributors, however unworthy, are easily accommodated, if not in one direction then in another. Everybody who styles himself "M. D." now finds abundant opportunity to air his views or solicit advice in a perplexing case and the result is a species of medical journalism as variegated and heterogeneous as the "Purple Cow" or "Lavender Pig" exemplars of general literature (?) that are constantly appearing upon the news-stands.

A critical examination of the contents of certain specimens of the class of medical journals to which we refer reveals on the part of their contributors an ignorance of the most commonplace facts of medical science that is simply deplorable and a sublime disregard for rules of syntax that is none the less deplorable, although infinitely more amusing. The conclusion to which the examiner inevitably comes is that the medical press of this country is in sad need of a censorship.

Dialect stories cannot compare, for bizarre spelling and phraseology, with the communications of certain medical gentlemen in distress over patients with "a touch of the consumption" or a complex of symptoms designated (shades of Charcot!) "a spell of the hysterics." From the intricacies of construction which characterise these writings, the Queen's English undergoes every variety of contortion until, finally, the very acme of elegant simplicity of diction is encountered in journals of the "correspondence" type. In these we find "Doc" So-and-so communicating his perplexities to 'Doc" Somebody-Else or bestowing upon him an infallible remedy for piles in exchange for a prompt and safe emmenagogue. All these little amenities are characterised by a style of diction that rivals the masterpieces of the "Ready Letter-writer" and demonstrate, if they do no more, that the lacteals of human kindness are never withered in the medical breast.

This is the class of writers about whom Dr. Holmes[1] says: "They go for weakness whenever they see it with stimulants and strengtheners, and they go for overaction, heat and high pulse and the rest, with cooling and reducing remedies." But their contributions can be of no positive value to scientific medical literature for the reason that they aid in no way toward a better understanding of disease processes. The movement which has set such a class of writings on foot is retrograde in that it tends to foster empirical methods of prosecuting a calling which now, more than at any other period of its existence, demands that its true representatives shall be scientists in the fullest sense of the word and not mere dispensers of powder and pellet.

The dignified attitude which medicine is beginning to assume among the sciences can never be maintained unless the writings which record its progress are of a quality to compare with those which mark the advances in the sister sciences.

We have gained so much of exact knowledge through pathological and experimental research that future contributions to current medical literature should be stripped almost entirely naked of theory and speculation, those barriers to scientific progress. To achieve this the medical writer must needs be an investigator, at least to the extent of finding out how far such views as he is about to put forth are substantiated by the facts of pathology. The neglect of such a preliminary step is today a tacit avowal of both laziness and ignorance, since our library facilities for such work are abundantly adequate. When the writer has before him all necessary material, he should aim, above all, at clearness and accuracy. His English should be terse, not oratorical and certainly not colloquial; it should be

1. The Poet at the Breakfast-table, Riverside Press edition, p. 121.

free from hybrid technical terms, especially those with Greek roots and Latin endings. Finally, his bibliography should be such as will bear verification, for there is nothing more maddening to the man who is searching the literature than to find reference after reference which resist all attempts at identification. The only accurate way to refer is to refer in full, even to the page number of every quotation.

In conclusion, we wish to allude to two faults which are rife enough among contributors to medical journals to be considered annoying, to say the least. The first is the eternal dragging in of the *ego*; and the second, an inordinate fondness for the sensational. With respect to the first, we can only say that in our opinion no man's writings will ever lose anything of dignity or worth by being, as far as possible, impersonal. As a sample of the second fault we need only call the reader's attention to the earlier literature of the *x*-ray. If he will but compare that with what represents the thoughts of men who have probed its usefulness to the bottom, the moral lesson will be obvious.

---

THE *Homeopathic Recorder*, for June, published by a drug firm, contains an article denunciatory of vaccination, entitled Vaccination, a fallacy—its compulsion, a crime! In the same issue the publishers print this card: "Vaccine points, always fresh at —— ——." Truly, belief and business do not run on the same narrow gauge track in homeopathy!

